# Understanding shallow soil moisture variation in the data-scarce area and its relationship with climate change by GLDAS data

**DOI:** 10.1371/journal.pone.0217020

**Published:** 2019-05-22

**Authors:** Jingping Zuo, Jianhua Xu, Weihong Li, Dongyang Yang

**Affiliations:** 1 Key Laboratory of Geographic Information Science (Ministry of Education), East China Normal University, Shanghai, China; 2 Research Center for East-West Cooperation in China, East China Normal University, Shanghai, China; 3 State Key Laboratory of Desert and Oasis Ecology, Xinjiang Institute of Ecology and Geography, Chinese Academy of Sciences, Urumqi, China; Columbia University, UNITED STATES

## Abstract

Quantitatively evaluating the spatiotemporal variation of soil moisture (SM) and its causes can help us to understand regional eco-hydrological processes. However, the complicated geographical environment and the scarce observation data make it difficult to evaluate SM in Northwest China. Selecting the Tarim River Basin (TRB) as a typical representative of the data-scarce area in Northwest China, we conducted an integrated approach to quantitatively assess the spatiotemporal variation of shallow soil moisture (SSM) and its responses to climate change by gathering the earth system data product. Results show that the low-value of SSM distributes in Taklamakan Desert while the high-value basically distributes in the Pamirs and the southern foothill of Tianshan Mountains, where the land types are mostly forest, grassland, and farmland. Annual average SSM of these three land types present a significant increasing trend during the study period. SM at 0–10 cm of all land types are positively correlated to precipitation in spring and autumn, and SM at 0–10 cm in the forest, grassland, and farmland are positively correlated with temperature in winter. SSM presents in-phase relation with precipitation whereas it presents anti-phase relation with temperature, with the significant resonance periods about 6–8 years and 2–3 years which mainly distribute from 1970s to early 1990s and 1960s respectively. The time lags of SSM relative to temperature change are longer than its lags relative to precipitation change, and the lags vary from different land types.

## Introduction

SM is an essential eco-hydrological factor, which contributes to the exchange of water and energy between earth's surface and the atmosphere [1, 2]. It accumulates the most information of the surface hydrological processes and changes the energy exchange between land-atmosphere by affecting the surface Alberto, the growth of vegetation, and evaporation [3]. SM is an essential factor affecting climate change and an important indicator reflecting the changes in surface hydrology [4]. Researchers have found that seasonal anomalies in soil moisture have vital influence on the seasonal variation of atmospheric circulation [5]. The effect of soil moisture on climate change even exceeds the effect of sea surface temperature in the circumstance of land [1]. For a specific region, the spatiotemporal variation of SM is mainly influenced by the climate change, hydrological cycle, and surface ecosystem. Moreover, SSM is an indicator of eco-hydrological processes and regional climate change. It is mainly affected by temperature, precipitation, sunlight, evaporation, and surface vegetation[6, 7]. Scholars have studied the change trend of SM and its relationship with atmospheric circulation, climate change, and NDVI. Precipitation and temperature are the two most important meteorological factors affecting the change of SM. [8–11]. However, there have relatively fewer researches about the spatiotemporal variation of SM and its response to the climate change under different land types, especially in Northwest China, a typical arid and semi-arid area in the continental interiors.

Climate of Northwest China is gradually becoming warmer and wetter with the global warming [12–15]. Under the impacts of regional and global climate variation, how does the SSM of different land types change? What is the spatiotemporal variation pattern of SSM? How does it respond to the climate change? It is not easy to reply to these questions thoroughly. It is difficult to answer these questions because of the complex geographic environment and scarce observation data in Northwest China.

We gathered the earth system data product to solve the problem of lacking observation data, including the SM data and the grid data of precipitation and temperature. The Global Land Data Assimilation System (GLDAS) combines satellite and surface-based meteorological observation data to provide reliable data for the research of SM [16, 17]. The GLDAS outputs exist estimated deviations and limitations and can hardly avoid the uncertainties of parameters and the atmospheric driving data, which will make influences on the research results [18–21]. However, there still have some studies acknowledged the availability of this dataset. Researchers used GLDAS data to conduct a lot of studies, which confirms the reliability of this dataset [22, 23]. The evaluation of soil data and temperature data of GLDAS reveal that this dataset has good reliability[24]. GRACE and GLDAS data was used to investigate the variation of terrestrial water storage in the Tianshan Mountains and its surrounding regions, the results indicated that GLDAS data have a good consistency and linear relationship with GRACE data [25]. In particular, SM data of GLDAS show good correlation and consistency with observation data, which is consistent with many studies [26–28].

**Fig 1:**
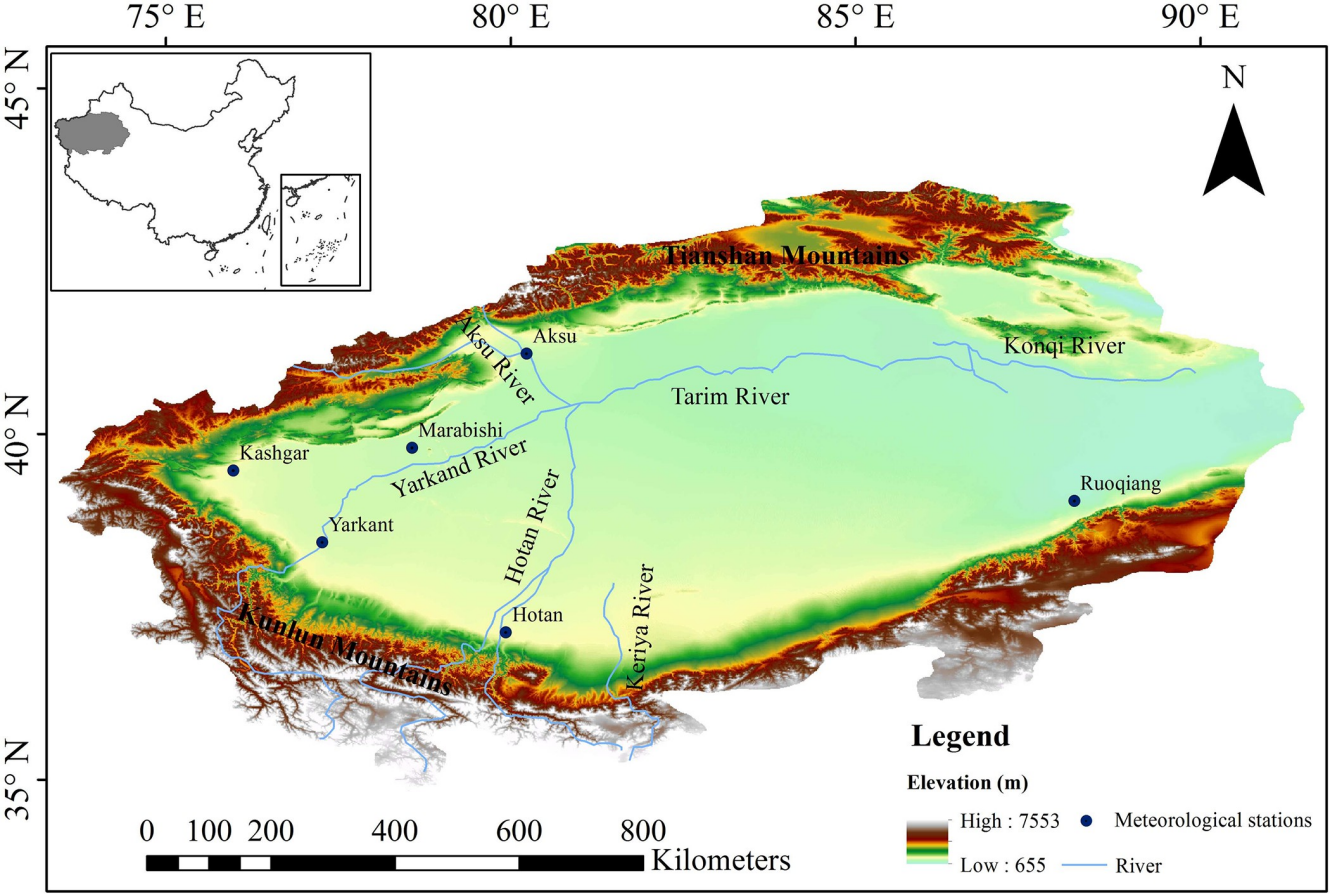
The study area. (The GTOPO30 DEM data are from USGS, https://earthexplorer.usgs.gov).

To understand the spatiotemporal variation of SSM and its relationship with precipitation and temperature under different land types in Northwest China, we selected the TRB as a typical representative of the data-scarce area in Northwest China (Fig 1). Based on the SM data of GLDAS and the monthly grid data of precipitation and temperature in China to investigate the spatiotemporal variation of SSM and its relationship with precipitation and temperature under different land types from 1961 to 2010 by using the Mann-Kendall trend test, Pearson correlation coefficient and the cross wavelet transform. Soil thickness not only reflects soil development, but also affects soil nutrients, water migration, and the growth of plant root. The average soil thickness in Xinjiang is about 68.9 cm [29]. Research indicated that the soil thickness of the Hapli-Gelic Camboso [30] in the Qilian country, Qinghai Province is about 48 cm [31]. Considering the mountainous with high altitude in the TRB where distributed frozen soil, we took the soil thickness of 48 cm as the reference for the study of SSM. GLDAS SM consists of four layers of 0–10 cm, 10–40 cm, 40–100 cm, 100–200 cm. Therefore, we selected the first two layers of GLDAS SM to investigate the variation of SSM in the TRB. In the following work, we defined the soil depth of 0–40 cm as the shallow soil layer, which means that the SSM refers the SM in the layer with the depth of 0–40 cm.

## Study area

A typical representative of the data-scarce area in Northwest China, the TRB locates in Xinjiang Province of Northwest China, and it is the largest inland river basin in China (Fig 1). This basin mainly consists of the Aksu River, Kashi River, Yarkand River, Hotan River, Kaidu River-Konqi River and other main tributaries as well as the mainstream of the Tarim River[32, 33]. The north of the TRB is the Tianshan Mountains, the southwest is the Pamirs and the Karakoram Mountains, the south is the Kunlun Mountain and the Altun Mountains, and the central region is the Taklimakan Desert, consequently generating a closed basin surrounded by high mountains that leads to the relative enclosed ecosystem[34]. This river basin belongs to the typical continental climate, with drought climate, rare precipitation, large amount evaporation, limited water resources, and its natural vegetation mainly distributes near the river[35]. Land types in the north belong to the mountain desert, grassland, and coniferous forest, with the relatively high vegetation coverage. The central region is mainly covered by the desert, its vegetation mainly distributes in the oasis agricultural district and the upper and middle reaches of the Tarim River. The vegetation coverage of the western Kunlun Mountains of the south is relatively high, which are mainly grassland and alpine vegetation [36–38]. Referring the relevant research literatures [39, 40], we mainly classify land types of TRB into five categories: desert, forest, grassland, farmland, and other land types.

## Materials and methods

### Materials

To investigate the spatiotemporal variation of SSM in the TRB, we used the monthly data of SM during the period from 1961 to 2010 with a spatial resolution of 0.25°×0.25°, which are obtained from the GLDAS-NOAH-2.0 dataset (https://hydro1.gesdisc.eosdis.nasa.gov/data/GLDAS/GLDAS_NOAH025_M.2.0/). The GLDAS SM data include four layers (i.e. 0–10 cm, 10–40 cm, 40–100 cm, and 100–200 cm). As defined above, the SSM refers the SM in the layer with depth of 0–40 cm, so we only used the data of two layers (i.e. the 0–10 cm and 10–40 cm). To evaluate the availability of GLDAS SM data, we used observation data of relative SM from 1992 to 2010, which were downloaded from China Meteorological Administration (http://data.cma.cn). The observation depth includes 10 cm, 20 cm, 50 cm, 70 cm, and 100 cm. In this research, we selected the 10 cm and 50 cm observation data to evaluate the GLDAS SM data at 0–10 cm and 10–40 cm. The distribution of observation stations is mapped in the Fig 1.

In order to show the response of SSM to climate change, we also used the monthly grid data of precipitation and temperature with the spatial resolution of 0.5°×0.5° in the same period, which are a subset of the grid dataset of ground surface precipitation and temperature gathered from the China Meteorological Administration (http://data.cma.cn).

To make sure the same spatial resolution with 0.25°×0.25° as the SM data, the precipitation and temperature data were resampled by using the bilinear interpolation method, and then use the grid points of SM data that within the areas of each land type to extract the values of precipitation and temperature. The GTOPO30 DEM (about 1 kilometer) is downloaded from USGS (https://earthexplorer.usgs.gov).

### Methods

This research used The Mann-Kendall test to examine the change trend of SSM and also used the Pearson correlation coefficient and cross wavelet transform to explore the relationship and time lags of SSM relative to the variation of precipitation and temperature.

#### Mann-Kendall test

The Mann-Kendall test was used to detect the trend in the interannual and seasonal variation of SSM in the TRB. The Mann-Kendall test is a non-parametric testing method, which is widely used to investigate the significance of the trend in climatic and hydrological time series[41, 42]. For a time series *Xt* = (*x*_1_,*x*_2_,⋯,*x*_*n*_), the statistic *S* of the Mann-Kendall test is expressed as follows[43]:

$$S=\sum_{i=1}^{n-1}\sum_{k=i+1}^{n}sgn(x_{k}-x_{i})$$ (1)

where, *X*_*k*_ and *X*_*i*_ are the annual values in years *k* and *i* respectively, *n* is the length of the data sample, *sgn* is symbolic function:

$$sgn(\theta )=\left\{ \begin{array}{l} 1\;(\theta >0)\\ 0\;(\theta =0)\\ -1\;(\theta <0) \end{array}\right.$$ (2)

when the length of data sample greater than or equal to 8, the statistic *S* is close to a normal distribution, its average value is zero, and the variance is:

$$Var(S)=\frac{n(n-1)(2n+5)-\sum_{i=1}^{n}t_{i}(i-1)(2i+5)}{18}$$ (3)

where *t*_*i*_ is the number of the i-th group data.

The standardized statistic *Z*_*c*_ is expressed as follows:

$$Z_{c}=\left\{ \begin{array}{l} \frac{S-1}{\sqrt{Var(S)}},S>0\\ 0\;,S=0\\ \frac{S+1}{\sqrt{Var(S)}},S<0 \end{array}\right.$$ (4)

the *Z*_*c*_ value is a trending statistic. If the *Z*_*c*_ is positive, it indicates that the tested sequence shows an increasing trend, and the negative value indicates a decreasing trend. If the absolute value of the *Z*_*c*_ is greater than 1.64 (the 95% confidence level), indicating that the trend of the sequence is significant. β represents the slope, which is commonly used to measure the magnitude and direction of the trend. The positive value represents rises and the negative value represents decline, the formula is:

$$\beta =Median\left(\frac{x_{i}-x_{j}}{i-j}\right),\forall j<i$$ (5)

#### Pearson correlation coefficient

We used Pearson correlation coefficient to measure the correlation of SSM with precipitation and temperature in the corresponding periods. For the data series *x*_1_,*x*_2_,⋯*x*_*n*_ and *y*_1_,*y*_2_,⋯*y*_*n*_ of the two variables *X* and *Y*, the formula is as follows[44]:

$$R=\frac{\sum_{i=1}^{n}\left(x_{i}-\bar{x})(y_{i}-\bar{y}\right)}{\sqrt{\sum_{i=1}^{n}(x_{i}-{\bar{x})}^{2}}\sqrt{\sum_{i=1}^{n}(y_{i}-{\bar{y})}^{2}}}$$ (6)

the range of the correlation coefficient is [–1, 1]. If *R* > 0, the two variables are positively correlated; if *R* = 0, the two variables are independent and have no correlation; if *R* < 0, it means the two variables are negatively correlated.

#### Cross wavelet transform

Cross wavelet transform (XWT) method effectively combines wavelet transform[45] and cross spectrum analysis, which can investigate the correlation of two time series in time-frequency domain from multiple time scales[46, 47].

We used the XWT method to investigate the multi-scale correlation of SSM with precipitation and temperature, as well as the time lags of SSM to the variation of precipitation and temperature.

The XWT of the two series *X* and *Y* are *W*_*X*_*(S)* and *W*_*Y*_*(S)*, respectively, then the XWT can be defined as:

$$W_{n}^{XY}(s)=W_{n}^{X}(s)W_{n}^{Y*}(s)$$ (7)

where *W*_*n*_^Y*^(s) denotes the complex conjugate of *W*_*n*_^*Y*^(s) and s is a time delay. The corresponding wavelet power spectral density is |*W*_*n*_^*XY*^(s)|. This value becomes larger means the more significant correlation between the two time series, and reflects both have a common high-energy. Significant test of continuous cross wavelet power spectrum, usually using the red noise standard spectrum[46, 48].

## Results and discussion

### The evaluation of availability for GLDAS SM data

To further evaluate the availability of GLDAS SM data in the TRB, we calculated the correlation coefficients of GLDAS SM data and observation data (Table 1). We selected the 10 cm and 50 cm observation data to evaluate the GLDAS SM data at 0–10 cm and 10–40 cm. The results of evaluation indicate that the GLDAS SM data present a relatively good correlation with observation data, which means the GLDAS SM data can reflect the temporal variations of SM and are applicable in the TRB. The results are consistent with the research of the assessment of SM data over China that GLDAS SM data have relatively good correlation with observation data and can better describe the seasonal and interannual variations of SM [28].

**Table 1:** The correlation coefficient of GLDAS SM data and observation data.

Category	Aksu	Marabishi	Hotan	Kashgar	Ruoqiang	Yarkant
**0–10 cm**	0.6679*	0.6440*	0.6162*	0.8257*	0.8401*	0.5232*
**10–40 cm**	0.6327*	06042*	0. 5179*	0.6857*	0.8327*	0.4412*

* Represents passed 0.05 significance test. We selected the observation data of each station, which have common periods with GLDAS SM data.

### The spatial patterns of SSM

**Fig 2:**
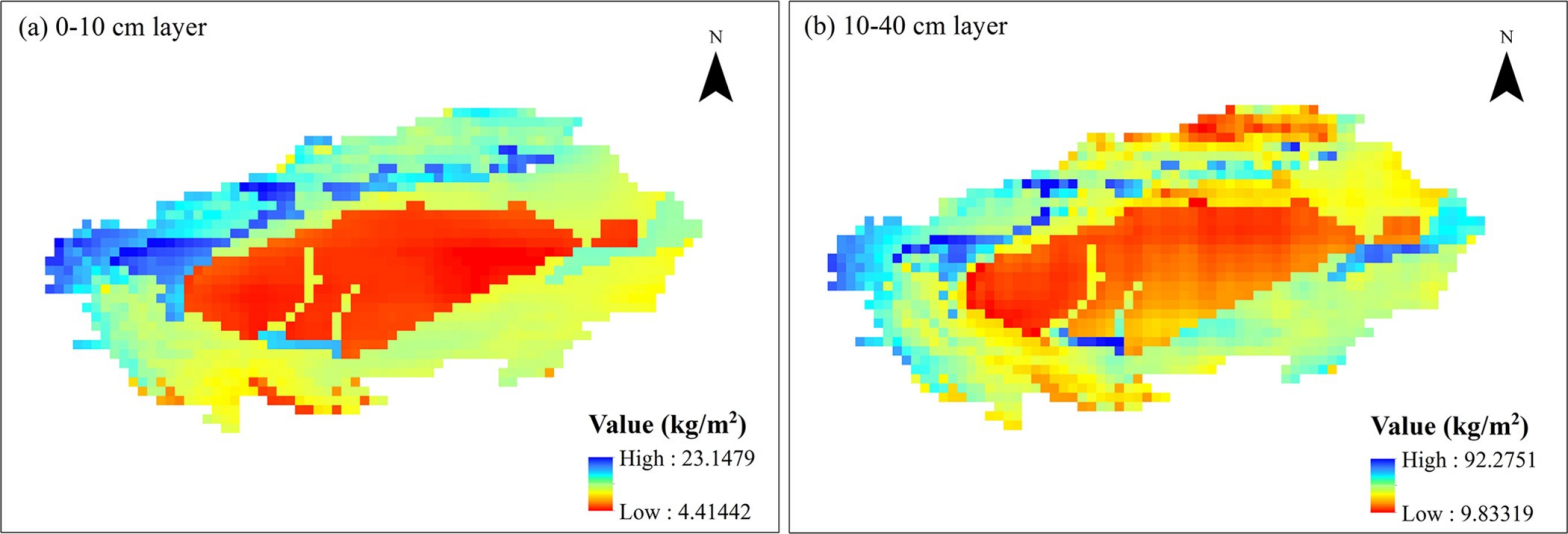
The spatial distribution of average SSM in the TRB from 1961 to 2010. (The average SSM data are extracted from the free shared data, GLDAS SM data, which are from the NASA Goddard Earth Sciences Data and Information Services Center.).

The SSM at 0–10cm and 10–40cm in the TRB are mapped to show their spatial patterns. We can see that the spatial distribution of SM at 0–10 cm (Fig 2A) and 10–40 cm (Fig 2B) in the TRB is basically consistent during the study period. The low-value of SSM distributes in the central regions while the high-value distributes in the Pamirs and the southern foothill of Tianshan Mountains with high altitude, and the land types are mainly forest, grassland, and farmland. These regions have relatively abundant precipitation, high vegetation cover, and relatively strong water storage capacity. The low-value of SSM mainly distributes in the Taklimakan Desert, with scarce precipitation, large amount evaporation, and strong water permeability.

### The interannual and seasonal variation of SSM

**Fig 3:**
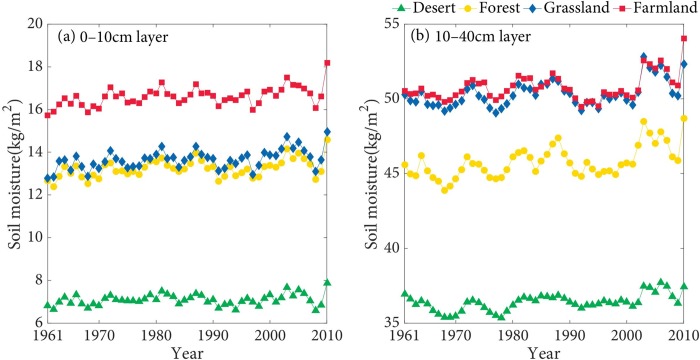
The interannual variation of the annual average SSM in the TRB from 1961 to 2010.

As can be seen from the Fig 3, annual average SM at 0–10 cm in the farmland of the TRB is considerably higher than that in other land types. Annual average SM at 0–10 cm in the forest only has small differences with the grassland, and annual average SM at 0–10 cm in the desert is the lowest among all land types (Fig 3A). Additionally, the differences of annual average SM at 10–40 cm between the farmland and grassland are relatively small, and annual average SM at 10–40 cm in the farmland and grassland are considerably higher than other land types. Similarly, annual average SM at 10–40 cm in the desert is the lowest among all land types (Fig 3B).

**Fig 4:**
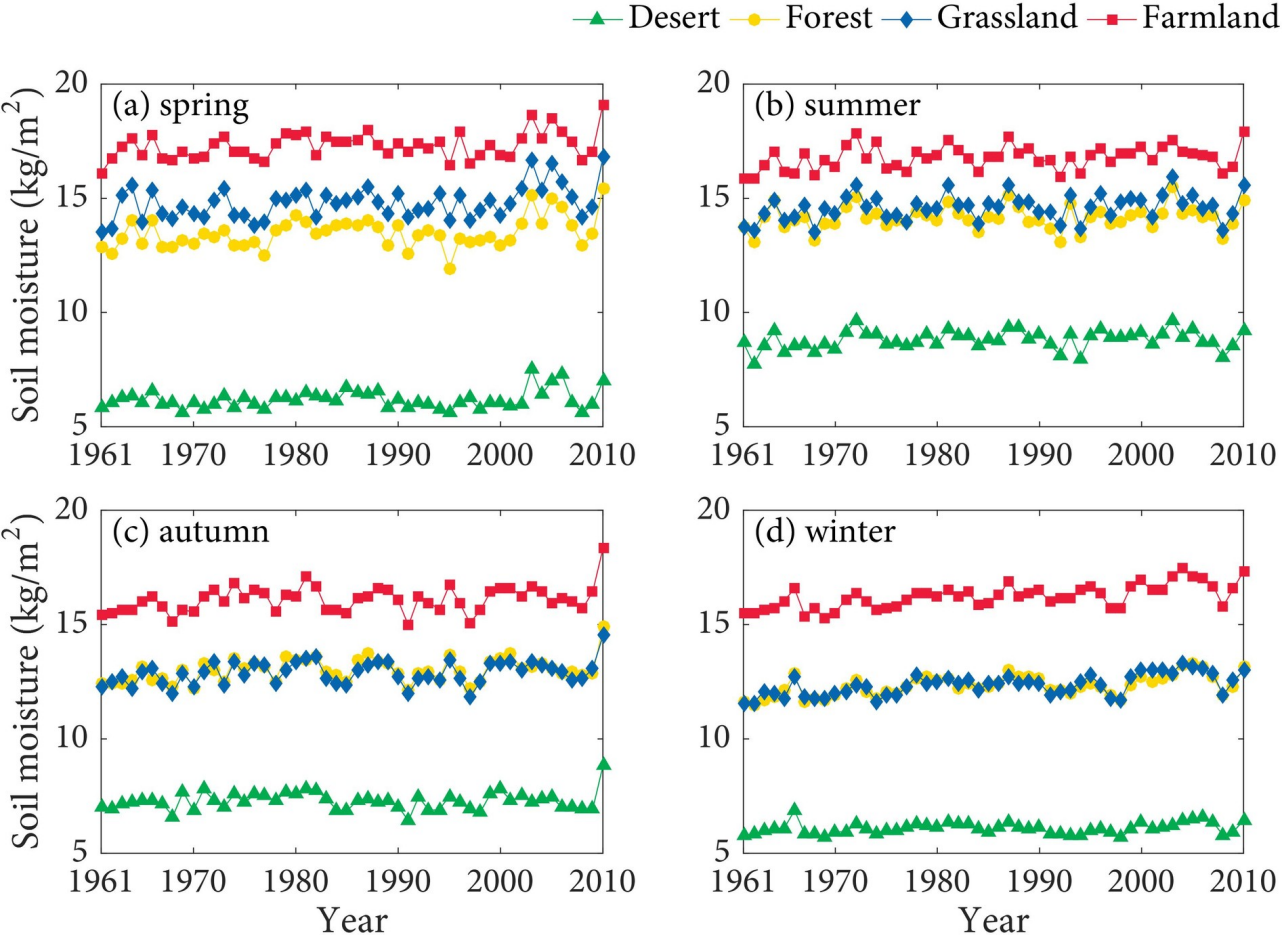
The seasonal variation of SM at 0–10 cm in the TRB.

**Fig 5:**
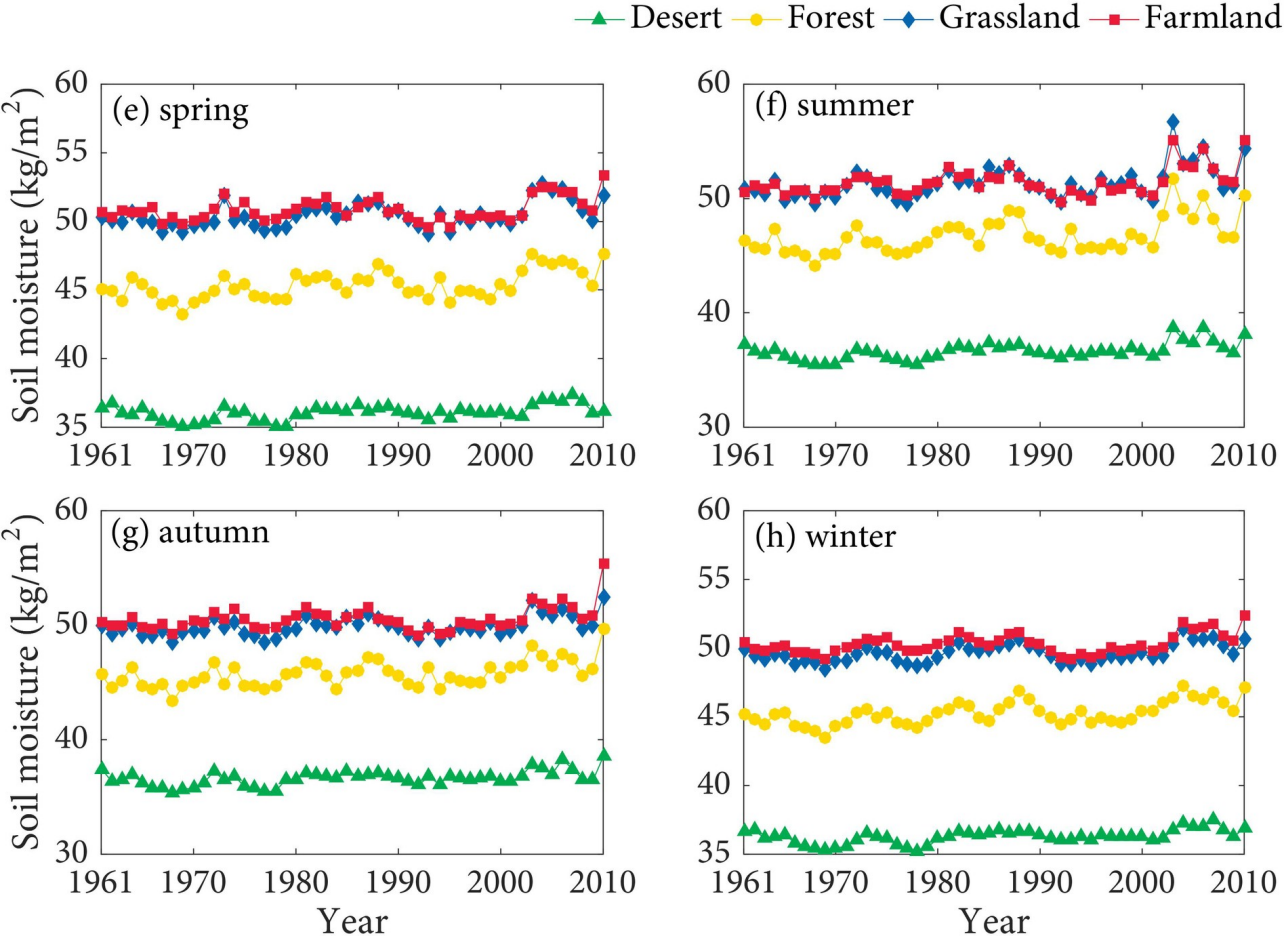
The seasonal variation of SM at 10–40 cm in the TRB.

As shown in the Figs 4 and 5, SM at 0–10 cm in the farmland is higher than that in other land types in the spring, summer, autumn, and winter. The seasonal differences of SM in the forest and grassland are relatively small. SM at 0–10 cm in the desert is the lowest. Besides, SM at 10–40 cm in the farmland and grassland has quite small differences, and both are significantly higher than that in other land types. SM at 10–40 cm in the desert is the lowest. SSM of the farmland in spring and summer are higher than that in other seasons, which are probably influenced by the agricultural irrigation and relatively rich precipitation in summer. The growth of spring wheat requires relatively abundant water, the experiment of the effect of Irrigation on the SM of spring wheat indicated that irrigation has obvious effect on SM [49]. Additionally, SSM in the forest, grassland, and desert are commonly highest in the summer. The main reason probably is that precipitation is the principal source of SSM [50], and precipitation is relatively abundant in summer.

### The change trend of SSM

The Mann-Kendall test is conducted to examine the change trend of SSM during the study period. The results are shown in Table 2 (under the confidence level of 0.05), it can be seen that the interannual variation of SSM in the forest, grassland, and farmland all present a significant increasing trend. For the seasonal variation, SM at 0–10 cm in the forest, grassland, and farmland show a significant increasing trend in the spring, summer, autumn, and winter, which are especially greater in the spring and winter. In addition, SM at 10–40 cm in the forest, grassland, farmland, and desert commonly present a significant upward trend in the spring, summer, autumn, and winter, which are especially greater in the summer. Studies indicate that the precipitation and temperature in the study area has a significant increasing trend during past several decades[51]. Precipitation is one important source of SSM [50], and the increases of temperature will cause the thawing of frozen soil and snow, which are another important source of SSM. So that the increases of SSM in the TRB are closely related to the climatic warming and wetting trend.

**Table 2:** The Mann-Kendall test of SSM.

Land types	Category	Z_c_		Slop	
		0–10 cm	10–40 cm	0–10 cm	10–40 cm
**Desert**	Year	1.4388	3.1954*	0.0044	0.0204
	Spring	0.4852	2.6600*	0.0018	0.0163
	Summer	1.7566*	3.3961*	0.0077	0.0244
	Autumn	0.1004	2.8441*	0.0003	0.0188
	Winter	1.8570*	3.0448*	0.0047	0.0169
**Forest**	Year	2.8273*	3.8813*	0.0127	0.0382
	Spring	2.2251*	3.3961*	0.0132	0.0345
	Summer	1.6845*	3.5630*	0.0059	0.0462
	Autumn	1.8905*	3.4129*	0.0102	0.0369
	Winter	4.2493*	4.1657*	0.0201	0.0324
**Grassland**	Year	3.5802*	2.9612*	0.0150	0.0240
	Spring	2.4258*	2.8273*	0.0181	0.0244
	Summer	1.9908*	2.9779*	0.0124	0.0347
	Autumn	1.7399*	2.5931*	0.0084	0.0205
	Winter	4.6509*	2.9110*	0.0224	0.0185
**Farmland**	Year	3.7307*	2.0578*	0.0145	0.0173
	Spring	1.9741*	1.6562*	0.0120	0.0136
	Summer	2.0243*	2.2920*	0.0111	0.0214
	Autumn	2.1079*	2.2083*	0.0105	0.0175
	Winter	5.3535*	2.4091*	0.0270	0.0181

* Represents passed 0.05 significance test.

### The simultaneous correlation of SSM with precipitation and temperature

Precipitation and temperature are often considered as the principal climatic factors that can affect the variation of SM in relevant studies [52–54]. In the following work, we mainly discuss the correlation of SSM with precipitation and temperature in the TRB.

The results of Pearson correlation coefficient are shown in Table 3. At the confidence level of 0.05, SM at 0–10 cm in the forest, grassland and desert all have a positive correlation with precipitation in spring, summer, and autumn during the same periods. Due to the relatively abundant precipitation and the relatively small evaporation in autumn, SM at 0–10 cm in the forest and grassland are positively correlated with precipitation in autumn, with the correlation coefficients of 0.5546 and 0.6664, respectively. SM at 0–10 cm in the farmland is positively correlated with precipitation in spring, autumn and winter, and its correlations are more significant in autumn, with a correlation coefficient of 0.6362. However, SM at 0–10 cm in the farmland has no obvious correlation with precipitation in summer. In addition, SM at 0–10 cm in the desert presents a positive correlation with precipitation in spring, summer, and autumn over the same periods, with correlation coefficients of 0.4688, 0.4740, and 0.3781, respectively. It is mainly for the reasons that the impact of precipitation on SSM in the desert is more direct and the increases of precipitation will affect the variation of SSM. The snowmelt in the spring will gradually gather in the middle area due to the terrain, which will make SSM increase to some extent.

**Table 3:** The correlation of SSM with precipitation and temperature.

Land types	Category	Precipitation		Temperature	
		0–10 cm	10–40 cm	0–10 cm	10–40 cm
**Desert**	Spring	0.4688**	0.2506	-0.0737	0.2413
	Summer	0.4740**	0.1660	-0.1070	0.2588
	Autumn	0.3781**	0.2695	0.0363	0.2701
	Winter	0.0006	-0.0634	0.1870	0.2717
**Forest**	Spring	0.4417**	0.2773	0.0014	0.1461
	Summer	0.3602*	0.2128	-0.1641	0.2475
	Autumn	0.5546**	0.4134**	0.1232	0.2879
	Winter	0.2487	0.1980	0.3932**	0.3973**
**Grassland**	Spring	0.3307*	0.1991	0.0506	0.1773
	Summer	0.4008**	0.2354	0.2110	0.2015
	Autumn	0.6664**	0.3086*	0.0931	0.2202
	Winter	0.1343	0.1665	0.3976**	0.2351
**Farmland**	Spring	0.4217**	0.2182	-0.1153	0.1827
	Summer	0.0724	0.1255	-0.3280*	0.0715
	Autumn	0.6362**	0.4741**	0.0539	0.2742
	Winter	0.3120*	0.1702	0.5079**	0.1708

* Represents passed 0.05 significant test

**represents passed 0.01significant test.

SM at 10–40 cm in the forest, grassland and farmland have a significant positive correlation with precipitation in autumn during the corresponding periods, with the correlation coefficients of 0.4134, 0.3086, and 0.4741, respectively (Table 3). We can illustrate that SSM in the TRB has relatively significant positive correlation with precipitation in autumn during the corresponding periods. However, the correlation between precipitation and SM at 10–40 cm in the desert is quite weak. In the Taklimakan Desert, the average annual precipitation is only about 50 mm, but its average evaporation is as high as approximately 1200 mm [55], which convert most precipitation to water vapor before it seeps into the deeper soil layers.

SM at 0–10 cm in the forest, grassland and farmland have a significant positive correlation with temperature in winter during the corresponding periods, with the correlation coefficients of 0.3932, 0.3976 and 0.5079, respectively (Table 3). The main reason is that the higher or lower temperature in winter influences the soil frozen status and hydro-thermal regime [50, 56]. However, the correlation between temperature and SM at 0–10 cm in the desert is unobvious. Moreover, SM at 0–10 cm in the farmland is negatively correlated with temperature in summer. For the reason of high temperature will result in the increases of potential evaporation which will reduce SSM.

For the simultaneous correlation between temperature and SM of 10–40 cm layer, only SM at 10–40 cm in the forest has a significant positive correlation with temperature in winter during the same periods. Comparing with the other land types, forest mainly distributes in the southern foothill of Tianshan Mountains and near the river, the influence of temperature change on the sunny slope and the river ice is relatively greater.

The relationships between SM, precipitation and temperature are quite different from the research in Eastern China [8]. The SM in Eastern China of different depths (0–200 cm) is positively correlated with precipitation in spring, summer and autumn. While the SM at 10–40 cm in the TRB is weakly correlated with precipitation. Moreover, the SM at 0–10 cm and 10–40 cm of Eastern China is negatively correlated with temperature in winter. However, the SM at 0–10 cm and 10–40 cm in the TRB is positively correlated with the temperature in winter.

From the results of Pearson correlation coefficient, we can find that SM at 0–10 cm of all land types is positively correlated to precipitation in spring and autumn, and SM at 0–10 cm in the forest, grassland, and farmland is positively correlated with temperature in winter.

### Multi-scale correlation and time lags

**Fig 6:**
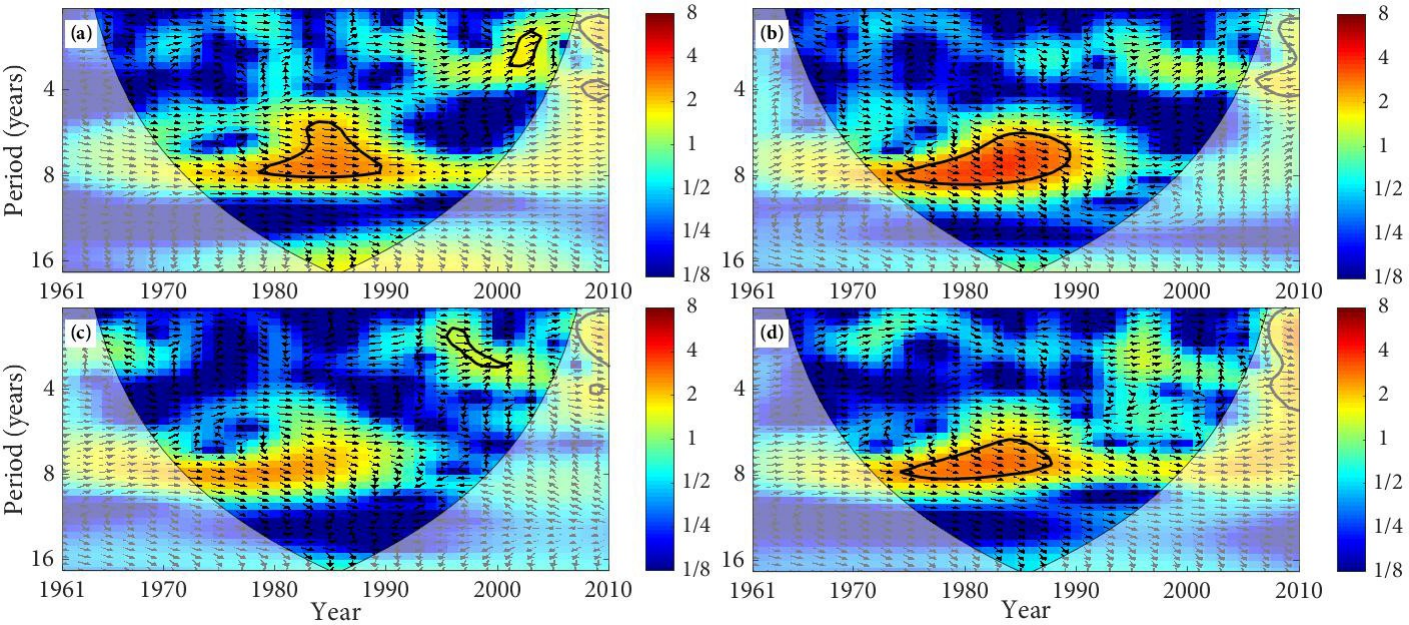
The cross wavelet transform of precipitation and SM (0–10 cm). (The area surrounded by the thick black line indicates the 5% significance level against red noise and the cone of influence (COI) with edge effects is shown as a lighter shade. Arrows presents the relative phase relationships between precipitation and SM, where the right direction means in-phase, the left direction means anti-phase, and up direction indicates that precipitation leading SM by 90°. The land types are (a) desert, (b) forest, (c) grassland, and (d) farmland.).

**Fig 7:**
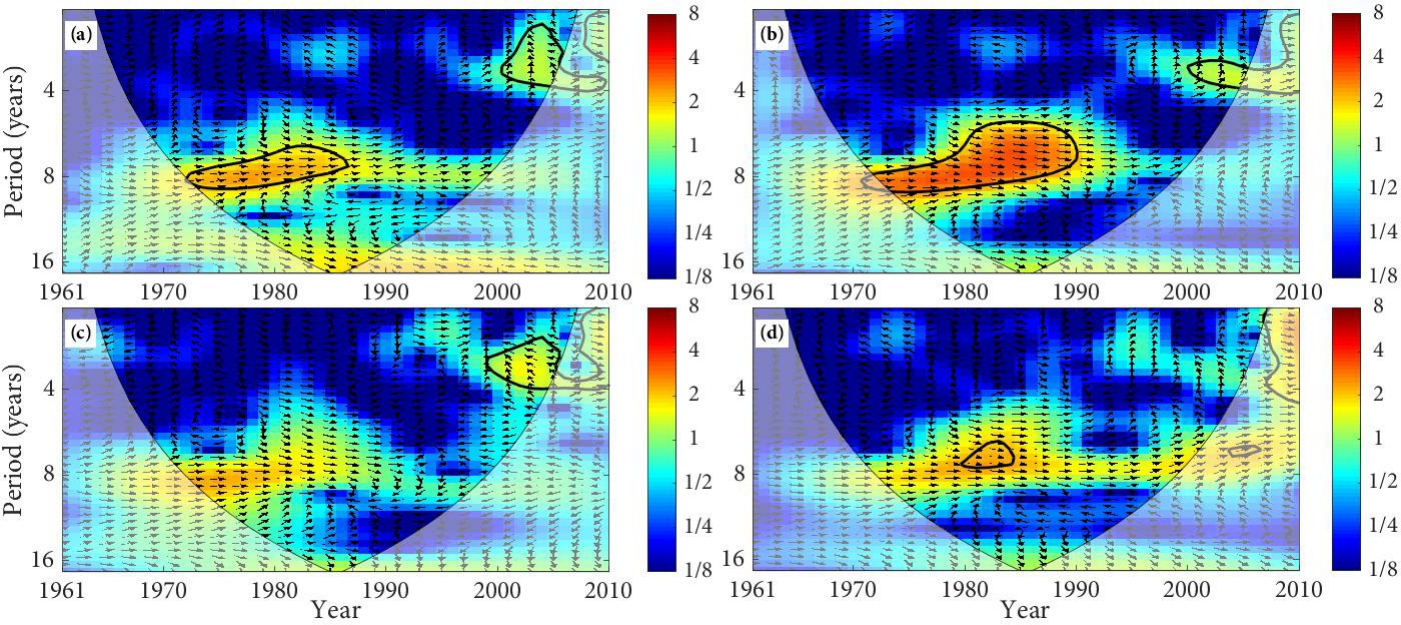
The cross wavelet transform of precipitation and SM (10–40 cm). (The area surrounded by the thick black line indicates the 5% significance level against red noise and the cone of influence (COI) with edge effects is shown as a lighter shade. Arrows presents the relative phase relationships between precipitation and SM, where the right direction means in-phase, the left direction means anti-phase, and up direction indicates that precipitation leading SM by 90°. The land types are (a) desert, (b) forest, (c) grassland, and (d) farmland.).

The XWT of precipitation and SSM is mapped in Figs 6 and 7 and Table 4. We notice that SM at 0–10 cm in the desert and precipitation was in-phase with significant common power of 6–8 year band from 1979 to1990. This in-phase relationship indicates that SM will increase with the increases of precipitation and decrease with the decreases of precipitation. Similarly, SM at 0–10 cm in the forest and farmland mainly presented an in-phase relationship with precipitation. However, SM at 0–10 cm in the grassland presents a significant in-phase relationship with precipitation from 1995 to 2001 (Fig 6). Moreover, SM at 10–40 cm in the desert, forest, and farmland has a significant in-phase relationship with precipitation. SM at 10–40 cm in the grassland has an in-phase relationship with precipitation from 1995–2001 (Fig 7). In the TRB, the principal resonant periods of precipitation and SSM in the desert, forest, and farmland are about 6–8 year, with an in-phase relation. The significant main resonant periods of precipitation and SSM in the grassland are about 2–3 years.

**Table 4:** The cross wavelet transform of SSM with precipitation and temperature.

Land types	Category	Period /years	Time range	Mean phase angle/°	Relation	Time lags/d
**Desert**	PRE·0–10	6–8	1979–1990	0.35°±0.11°	in-phase	0.3549
	PRE·10–40	6–8	1972–1987	0.07°±0.12°	in-phase	0.0710
	TEM·0–10	2–3	1961–1969	-2.04°±0.81°	anti-phase	2.0683
	TEM·10–40	2–3	2002–2006	1.29°±0.36°	in-phase	1.3079
**Forest**	PRE·0–10	6–8	1974–1990	0.86°±0.13°	in-phase	0.8719
	PRE·10–40	6–8	1972–1990	0.35°±0.06°	in-phase	0.3549
	TEM·0–10	2–3	1964–1969	-1.76°±0.05°	anti-phase	1.7844
	TEM·10–40	2–3	1964–1966	-3.12°±0.03°	anti-phase	3.1633
**Grassland**	PRE·0–10	2–3	1995–2001	0.28°±0.13°	in-phase	0.2839
	PRE·10–40	2–3	1999–2006	-2.45°±0.03°	anti-phase	2.4840
	TEM·0–10	2–3	1964–1969	-2.42°±0.17°	anti-phase	2.4536
	TEM·10–40	2–3	2002–2006	-2.27°±0.07°	anti-phase	2.3015
**Farmland**	PRE·0–10	6–8	1974–1987	0.77°±0.11	in-phase	0.7807
	PRE·10–40	6–8	1980–1885	0.24°±0.04°	in-phase	0.2433
	TEM·0–10	2–3	1995–1998	-2.97°±0.04°	anti-phase	3.0113
	TEM·10–40	2–3	2005–2008	-2.80°±2.39	anti-phase	2.8389

Note: PRE·0–10 presents the cross wavelet transform between annual average SM of 0–10 cm layer and auunal average precipitation, PRE·10–40 presents the cross wavelet transform between annual average SM of 10–40 cm layer and annual average precipitation. TEM·0–10 presents the cross wavelet transform between annual average SM of 0–10 cm layer and annual average temperature, TEM·10–40 presents the cross wavelet transform between annual average SM of 0–10 cm layer and annual average temperature.

**Fig 8:**
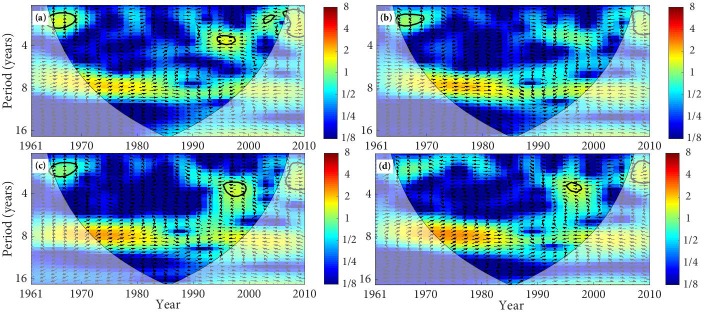
The cross wavelet transform of temperature and SM (0–10 cm). (The area surrounded by the thick black line indicates the 5% significance level against red noise and the cone of influence (COI) with edge effects is shown as a lighter shade. Arrows presents the relative phase relationships between temperature and SM, where the right direction means in-phase, the left direction means anti-phase, and up direction indicates that temperature leading SM by 90°. The land types are (a) desert, (b) forest, (c) grassland, and (d) farmland).

**Fig 9:**
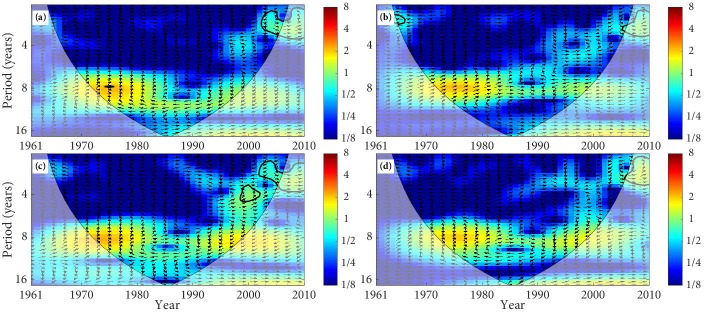
The cross wavelet transform of temperature and SM (10–40 cm). (The area surrounded by the thick black line indicates the 5% significance level against red noise and the cone of influence (COI) with edge effects is shown as a lighter shade. Arrows presents the relative phase relationships between temperature and SM, where the right direction means in-phase, the left direction means anti-phase, and up direction indicates that temperature leading SM by 90°. The land types are (a) desert, (b) forest, (c) grassland, and (d) farmland).

The XWT of temperature and SSM in the TRB is mapped in Figs 8 and 9. SM at 0–10 cm in the desert has a significant anti-phase relationship with temperature from 1961 to 1969 (Fig 8). This anti-phase relationship indicates that the SM will decrease with the increases of temperature and it will increase with the decreases of temperature. SM at 0–10 cm in the forest, grassland, and farmland all present an anti-phase relationship with temperature. In addition, SM at 10–40 cm in the desert, forest grassland, and farmland have an anti-phase relationship with temperature (Fig 9). We can find that the principal resonant periods of temperature and SSM in the TRB are about 2–3 year, with the anti-phase relation (Table 4).

The phase angle of XWT can figure out the time lags between tow time series. As can be seen from Table 4, SSM of the different land types have different time lags compared with the variation of precipitation and temperature. The time lags of the SSM relative to the temperature change is significantly longer than its time lags relative to the precipitation change, and the time lags vary from different land types. SM at 0–10 cm and 10–40 cm show different time lags compared with the precipitation change, with the lags of about 0.28d–0.87d and 0.07–0.35d, respectively. SM at 0–10 cm and 10–40 cm also present different time lags compared with the temperature change, with the lags of about 1.78d–3.01d and 1.30d–3.16d, respectively. The variation of SSM in the TRB is closely related to climate change. Precipitation is a principal and direct sources of SSM, which means the impact of precipitation on the variation of SSM is more direct. However, temperature affects the change of SM by affecting the soil freeze-thaw process and the evaporation of the surface soil layer [57–59]. Therefore, the time lags of SSM relative to temperature changes are longer than its time lags relative to precipitation change.

The results of XWT reveal that the significant correlation between precipitation and SSM mainly distributes in the periods from the 1970s to the early 1990s, and the significant correlation between temperature and SSM mainly distributes in the periods about the 1960s. It is basically consistent with the researches about the variation of drought and waterlogging. The drought of the TRB presented serious in the 1960s, and the transition periods from drought to waterlogging were about the 1970s to the 1980s, while the drought conditions significantly declined in the mid-to-late 1980s [60, 61].

The main results of the XWT show that there is a relatively strong link between precipitation and SSM, and it is mainly the in-phase relation, while temperature and SSM is mainly the anti-phase relation, and the link is relatively weak. The in-phase relationship indicates that SM will increase with the increases of precipitation and decrease with the decreases of precipitation, and the anti-phase relationship indicates that the SM will decrease with the increases in temperature and it will increase with the decreases of temperature. We can find that the variation of SSM in the TRB is more influenced by precipitation, and temperature is less effective in affecting of SSM change [10]. The time lags of SSM to temperature changes are longer than its time lags relative to precipitation, and the lags vary from different land types, which are different from previous researches [8, 10, 18, 50].

## Conclusion

We investigated the spatiotemporal variation of SSM in different land types and its responses to the climate change in the TRB based on the GLDAS SM data and the grid data of precipitation and temperature in China. This research used the Mann-Kendall test to examine the change trend of SSM, and also used the Pearson correlation coefficient and cross wavelet transform to explore the relationship and time lags of SSM relative to the variation of precipitation and temperature. The main conclusions are as follows: (1) the low-value of SSM distributes in the Taklamakan Desert, whereas the high-value of SSM mainly distributes in Pamirs and the southern foothill of Tianshan Mountains, where the land types are mainly forest, grassland, and farmland. (2) Annual average SSM and seasonal SSM of the forest, grassland, and farmland all present a significant increasing trend during the study period. (3) SM at 0–10 cm of all land types are positively correlated to precipitation in spring and autumn, and SM at 0–10 cm in the forest, grassland, and farmland are positively correlated with temperature in winter. (4) SSM presents in-phase relation with precipitation whereas it presents anti-phase relation with temperature, with the significant resonance periods, with the periods about 6–8 years and 2–3 years respectively. (5) The significantly related periods of SSM and precipitation are mainly from1970s to early 1990s, and its significantly related periods with temperature are mainly around 1960s. (6) The time lags of SSM relative to temperature change are longer than its time lags relative to precipitation change, and it varies from different land types.

## Supporting information

S1 Data SoilMoist data. (7Z)
